# Substantial psychosocial impairments in patients with chronic urticaria are associated with delayed referral to urticaria centers, non-academic treatments, and dietary changes 

**DOI:** 10.5414/ALX02554E

**Published:** 2025-02-10

**Authors:** Julia Zarnowski, Paula Kage, Regina Treudler

**Affiliations:** 1 Department of Dermatology, Venereology and Allergology, University Hospital Leipzig, Leipzig,; 2 Dermatological practice, Dr. med. Paula Kage, Freiberg, and; 3 Institute for Allergology, Charité – Universitätsmedizin Berlin, Corporate Member of Freie Universität Berlin and Humboldt-Universität zu Berlin, Berlin, Germany

**Keywords:** chronic urticaria, burden of disease, quality of life, anxiety, depression, psychosocial impairment

## Abstract

Background: Chronic idiopathic urticaria (CIU) is common in allergological practice. Although therapeutic options have improved in the past decade, patients still suffer from a significant burden of disease and are often treated insufficiently. Objective: We aimed at analyzing the psychiatric comorbidities, social impairments, and treatment gap in a real-world setting. Materials and methods: Adult patients with CIU were investigated for demographical data, medical history, and psychosocial burden. Validated questionnaires were used to assess urticaria activity, control of disease, quality-of-life impairment, and psychiatric comorbidities. Results: 82 patients (78% female; 47.5 ± 14.8 years) were included. 65.9% had insufficient disease control, 11% reported on prior self-medication with drugs, 19.5% were seeking help from non-academic medicine, and 54.9% tried a change of diet. The use of non-academic treatment was significantly associated with higher disease activity. Self-initiated dietary changes were significantly associated with less control of disease. Delayed referrals to a urticaria-specialized center were significantly linked to self-reported psychiatric diseases, self-medication with drugs and self-initiated dietary changes. Conclusion: Our data show an unsatisfactory control of CIU in many patients and substantial psychosocial impairments which are also associated with a delayed referral to urticaria centers, self-initiated non-academic treatments, and dietary changes.

## Introduction 

Chronic idiopathic urticaria (CIU) is a mast-cell driven disease characterized by recurring wheals and/or angioedema over a period of > 6 weeks, and the estimated global lifetime prevalence is ~ 1.4% with females being affected up to twice as often [1, 2, 3]. 

CIU can be aggravated by additional (physical) trigger factors such as temperature, pressure, stress, drugs, or infections. The occurrence of CIU with angioedema or physical triggers is associated with a longer disease duration and worse therapeutic response to antihistamines [1, 2, 4]. Disease duration can vary considerably between patients, and a spontaneous remission in up to 55% of patients within 5 years is reported, but it often takes > 1 year before appropriate clinical management is established [1, 5, 6, 7]. 

Due to the sudden and unpredictable symptoms that can occur at any time of the day (the burdening pruritus, disturbed sleep quality, and disfiguring angioedema) CIU has a huge emotional, social, and psychological impact on patients’ lives. Thus, the incidence of mental disorders as well as impairment of quality of life and sexual functioning in chronic urticaria patients has recently gained attention [8, 9, 10]. 

Psychiatric comorbidities are described in up to 50% of all CIU patients, with depression, anxiety, and somatoform disorders being most commonly reported [1, 3, 7, 9, 11, 12]. 

Multiple studies highlight the negative impact of CIU on sleep quality and cognitive functioning, also a reduced work performance and lower productivity with increased sick days and absences in children and adults suffering from CIU was reported [7, 13, 14, 15, 16, 17]. Other restrictions encountered by CIU patients are reported in daily activities, sports, social interactions, romantic relationships, and sexual functioning [7, 16, 18, 19]. In a study by Sanchez-Diaz et al. [10] including 75 CIU patients, sexual dysfunction was detected in over half of all studied patients, which showed a significant association with insufficient control of disease. Similarly, Ertas et al. [18] reported on sexual dysfunction in 67.9% of female CIU patients, which was associated with higher disease activity, anxiety, depression, and fatigue as well as poor control of disease. 

Previous data from German CIU patients have indicated a tremendous gap in CIU care, as over 50% of patients report to receive no adequate, effective, or satisfying treatment, and 67.5% experience frequent depressive symptoms [20]. 

Our aim was to evaluate the psychosocial burden, psychiatric comorbidities, and CIU care in a real-world setting of patients presenting to our tertiary referral center at University Hospital Leipzig, Department of Dermatology, Venereology and Allergology. 

## Materials and methods 

### Study design and ethics statement 

In this monocentric, questionnaire-based study we included adult patients with CIU who were referred to our GA2LEN urticaria center of reference and excellence (UCARE) at the Department Of Dermatology, Venereology and Allergology, University of Leipzig, Germany. Written consent was obtained from all participants. This study complies with ethical guidelines and received approval by the ethical board of the Medical Faculty of the University of Leipzig (Vote Nr.: 459/19-ek). 

### Questionnaires 

We used in-house questionnaires to collect demographic data, medical history and course of urticaria, comorbidities, and received or self-initiated treatment. To evaluate the burden on relationships and occupational disease a 5-item Likert Scale (none, mild, moderate, severe, very severe) was used. 

Validated questionnaires were used to assess control of disease, disease activity, quality-of-life impairment, and psychosocial outcome parameters: The Urticaria Control Test (UCT) aims to assess disease control over a recall period of 4 weeks. The UCT consists of 4 questions, which can be scored individually from 0 (strongly impaired/no control) to 4 (no impairment/good control), reaching a total score of 0 – 16. A cut-off value of < 12 points corresponds to poorly controlled disease [21]. 

The Urticaria Activity Score (UAS7) is used for 7 consecutive days to quantify disease activity. Patients rate the two main symptoms pruritus and wheals from 0 (none) to 3 (intense) once per day. After 7 days, the sums are added to give a total score ranging from 0 to 42 [22]. The UAS7 total score can be related to the weekly urticaria activity using following score ranges: 0 (urticaria free), 1 – 6 (well controlled); 7 – 15 (mild), 16 – 27 (moderate), and 28 – 42 (severe) [23, 24]. 

The Chronic Urticaria Quality Of Life Questionnaire (CU-Q2oL) is another patient-reported outcome tool that assesses the impairment on quality of life in different aspects, such as physical functioning, sleep, social relationships, mood/mental state, eating and choice of clothing, aesthetic impairment or embarrassment over the past 14 days. Patients rate 23 items on a Likert scale ranging from 1 (not at all) to 5 (very much). All items are added to a total score, which is transformed linearly to a 0 (no impairment) to 100 (significant impairment) scale [25]. 

The German version of the Centre for Epidemiologic Studies Depression Scale (CES-D) was used to assess depressive symptoms over the last 7 days. 20 items inquiring for depressive symptoms are rated on a Likert scale ranging from 0 (rarely/none) to 3 (mostly/all the time). Four items have an inverse rating and serve as control of correct understanding and exclusion of false statements. The total score can range from 0 – 60 and a cut off value of ≥ 23 is regarded as indicative for a major depression [26, 27, 28]. 

The Hospital Anxiety and Depression Scale (HADS) consists of 14 items, of which 7 relate to anxiety symptoms or depressive symptoms, respectively. Each item is rated from 0 (none) to 3 (pronounced feelings of, e.g., joylessness or fear) over a recall period of 1 week. A total sum is calculated individually for anxiety and depression and can range from 0 to 21. A sum score up to 7 is unsuspicious; a score between 8 and 10 indicates a moderate degree, and values ≥ 11 a pronounced degree of anxiety or depression [29]. 

Lastly, we used a German version of the established self-report questionnaire Generalized Anxiety Disorder-7 (GAD-7) to screen for anxiety. The GAD-7 (covering symptoms of generalized anxiety disorder, panic disorder, social anxiety disorder, and post-traumatic stress disorder) consists of 7 items asking patients how often, they were bothered by each symptom during the last 4 weeks. The score reaches from 0 to 3 points (0: not at all, 1: several days, 2: more than half of the days, 3: nearly every day) with a maximum score of 21. A total score of ≥ 10 indicates moderate anxiety symptoms, and a cut-off point ≥ 15 is indicative of severe anxiety symptoms [30]. 

### Statistical analysis 

Statistical analysis was performed with IBM SPSS Statistics, Version 29.0. Descriptive results were shown as numbers and percentages, means and standard deviations. A p-value of < 0.05 was considered statistically significant. Mann-Whitney U test was used to evaluate group differences, and Spearman’s rank correlation coefficient was used to measure the strength of associations. 

## Results 

### Patient characteristics 

82 patients (22% male; 78% female), aged 47.5 ± 14.8 years were included. Results of validated questionnaires are shown in Table 1. 

Atopic diseases were present in 23/82 patients (28%), most commonly allergic rhinitis (20/23; 87% of all atopic individuals), followed by allergic asthma in 8/23 (34.8%), and atopic dermatitis in 6/23 (26.1%). Autoimmune comorbidity was reported in 25/82 patients (30.5%) with Hashimoto’s thyroiditis being most common (14/25; 56.0% of all autoimmune comorbidities), followed by rheumatoid arthritis (4/25; 16.0%), vitiligo (3/25; 12.0%), inflammatory bowel disease, and type I diabetes (2/25; 8.0%, respectively), and lastly Basedow’s/Grave’s disease, alopecia, Hailey-Hailey disease, autoimmune hepatitis, multiple sclerosis (1/25; 4.0%, respectively). 

### Validated questionnaires 

Detailed data are given in Table 1. The UCT showed a good control of disease in only 31.4% (score ≥ 12). The mean UAS indicated severe urticaria activity and CU-Q2oL showed a significant burden of disease. Using CES-D, 23.2% reached a critical cut-off score of ≥ 23, indicating major depression. 57.3% of patients showed unsuspicious values (score ≤ 7) at HADS-Anxiety questionnaire, while 18.3% had a moderate and 20.7% had a severe degree of anxiety symptoms. In 25.6%, GAD-7 showed at least moderate anxiety (score ≥ 10) and in 11% severe anxiety symptoms (score ≥ 15). HADS-Depression was unsuspicious in 64.6%, while 15.9% showed a moderate and 15.9% a severe degree of depression. 

### Course of disease and treatment 

45/82 (54.9%) reported to have noticed trigger factors leading to an exacerbation of urticaria symptoms, most commonly heat (21/45; 46.7%), pressure (21/45; 46.7%), stress (25/45; 55.6%), infections (12/45; 26.7%), non-steroidal anti-inflammatory drugs (6/45; 13.3%), and cold (5/45; 11.1%). The majority of these patients (36/45; 80%) reported multiple triggers. 54.9% reported to additionally suffer from angioedema. 

Mean age at first symptoms was 40.5 ± 15.2 years with a mean disease duration of 6.1 ± 6.7 years, 40.2% (33/82) suffering ≥ 5 years, of whom one third (11/33; 33.3%) had a symptomatic CIU for ≥ 15 years. 

28% reported to have been treated at least once due to their CIU in an emergency department, 11% even multiple times. The time span between first symptoms and first presentation in a UCARE specialized clinic was 3.8 ± 6.4 years. A longer disease duration was significantly associated with a delayed referral to a specialized UCARE center (p < 0.001). 

With regard to treatment, 80.5% (66/82) received antihistamines, and 43.9% (36/82) were treated exclusively with antihistamines. 47.6% (39/82) received treatment with omalizumab and 8.5% (7/82) received another treatment (e.g., ciclosporin and/or systemic corticosteroids). A significant negative correlation was seen between sole antihistamine therapy and control of disease (UCT: p = 0.001) and positive correlation with disease activity (UAS7: p = 0.016). 

Treatment with omalizumab was significantly associated with a longer disease duration (p < 0.001), lower disease activity (UAS7: p = 0.037), and higher control of disease (UCT: p = 0.009). 

11% reported on prior self-medication with drugs (alcohol, nicotine, or marihuana) due to CIU symptoms, and 19.5% reported to have sought help from non-academic medicine, most commonly naturopathy, homeopathy, or acupuncture. 54.9% tried a change of diet, mostly (37/45; 82.2%) without prior recommendation by a physician. The use of non-academic treatment was significantly associated with higher disease activity (UAS7: p = 0.022), and self-initiated dietary changes were significantly associated with higher quality-of-life impairment (CU-Q2oL: p = 0.003) and less control of disease (UCT: p = 0.007). 

A significant correlation was seen between delayed referral to a urticaria-specialized center and self-reported psychiatric diseases (p = 0.022), psychiatric inpatient stays (p = 0.008), self-medication with drugs (p = 0.013), and self-initiated dietary alterations (p = 0.002). 

### Psychosocial burden and quality-of-life impairment 

41.5% (34/82) reported to be diagnosed with a psychiatric disorder, with depression being the most common in 76.5% (26/34), followed by sleep disorder in 35.3% (12/34), anxiety disorder in 26.5% (9/34), obsessive-compulsive disorder in 8.8% (3/34), personality or eating disorder in 5.9% (2/34, respectively), and addiction or phobia in 2.9% (1/34; respectively). 

In regard to suicidal ideation, 14.6% (12/82) reported to have had either thought about (12/12), planned (2/12), or attempted (1/12) suicide. Patients with a poor control of disease showed a significant association with self-reported suicidality in the past 12 months (UCT: p = 0.015). 

A positive correlation could be detected between psychiatric comorbidities and urticaria-related quality-of-life impairment (CU-Q2oL: p = 0.003). Self-reported psychiatric diagnoses as well as suicidal ideation were significantly associated with all psychosocial scores (GAD7, CES-D, HADS-A and HADS-D: p < 0,001, respectively). 

62.2% (51/82) reported a burden on romantic relationships due to their CIU, with 30% experiencing severe to very severe impairment. 81.7% reported a burden of CIU on working life, with 37.8% being severely to very severely burdened. Higher disease activity (UAS7) was significantly associated with higher perceived burden on occupational (p = 0.002) and romantic life (p < 0.001). Patients who were burdened in romantic and occupational life showed a significant positive correlation with GAD7 (p = 0.001 and p = 0.003), HADS-A (p < 0.001, respectively), HADS-D (p < 0.001 and p = 0.002), and CES-D (p = 0.002 and 0.004). Also, a reported impairment of romantic (p < 0.001) and occupational (p = 0.03) life was significantly associated with a self-reported suicidal ideation. No significant differences between the antihistamine-only versus omalizumab-treated group were detected in regard to all psychosocial scores (GAD7: p = 0.434; CES-D: p = 0.575; HADS-A: p = 0.471; HADS-D: p = 0.506). The parameters age, duration of disease and presence of angioedema showed no significant associations with psychosocial scores. 

## Discussion 

This monocentric investigation in CIU patients detected a frequent unsatisfactory control of CIU and substantial psychosocial impairments. 

Validated questionnaires showed depression in up to 30% and anxiety in up to 40% of patients. Additionally, suicidal ideation was present in almost 15%. 

Psychiatric comorbidities as well as suicidal ideation were also associated with a worse control of urticaria, as measured with UCT. 

Severe impairment of romantic and work life, as perceived by over 1/3 of our patients, was significantly associated with psychiatric comorbidities. There was also a significant association between delayed referral to a tertiary center and psychiatric comorbidities. 

About 20% of our patients had been seeking relief of their CIU symptoms by trying non-academic, alternative medical approaches, self-medication with drugs, or non-indicated and unsupervised dietary alterations. This complies with another nationwide German study that has reported dietary alterations in 42.1% of surveyed CIU patients, with 33.1% additionally reducing sports and 19.8% minimizing social activities due to their CIU symptoms [20]. Various other studies have also highlighted the significant impairment of CIU on daily life and social interactions as well as dietary changes [1, 11, 16, 20, 31]. 

Simultaneously, our data indicate a lower disease activity and better control of disease in patients treated with omalizumab compared to patients receiving antihistamines only. 

A review by Gonçalo et al. [7] shows that patients have to wait up to 4 years to receive a correct diagnosis, leading to a significant delay of treatment and pre-existing German data confirm a tremendous treatment gap in CIU [3, 7, 20, 32, 33]. As an example, an evaluation of a German statutory health insurance database showed that over 50% of all CIU patients had no recorded CIU treatment, and even though dermatologists were most commonly the ones prescribing biological treatments, they were consulted in only 6.6% of all CIU cases [32]. In a study conducted by Weller et al. [34], 41 CIU specialists from German tertiary care centers were interviewed concerning their experience in CIU care. Here, 61.0% reported on difficulties in patient care between specialized centers and practice-based physicians, which, for instance, became evident in the discontinuation of previously started medication. The study deduces that selected CIU patients, especially with antihistamine-refractory disease, might benefit from a referral to specialized urticaria centers [34]. Another nationwide German survey stated that although over 50% of participants reported to be dissatisfied with their treatment, only 6.8% were referred to a specialized urticaria center. In the same survey, 47.6% received topical treatment, although no evidence or guideline recommendation in national and international guidelines exists for its effectiveness in CIU treatment [1, 20]. 

The ATTENTUS study with over 9,000 participants showed that 60% of CIU patients were not treated by a physician although being symptomatic. Around 54% of regularly symptomatic patients who had stopped seeing their treating physician stated that they did not expect to receive help by their doctor, and 74% of patients with a disease duration of ≥ 15 years stopped seeking medical treatment altogether [33]. 

A significant component in managing CIU with its accompanying psychosocial impairments and psychiatric comorbidities is an early, correct diagnosis, and consequently the initiation of appropriate, effective therapies [1, 3, 35]. In this context, other authors highlight the treatment gap and conclude that CIU patients in Germany are highly burdened and their disease is often insufficiently controlled [20, 32, 33, 34]. This is in accordance with our study, as our patient cohort displays an unsatisfactory control of CIU and substantial psychosocial impairments which are additionally associated with a delayed referral to urticaria centers, self-initiated alternative treatments, and dietary changes. Limitations of our study include the monocentric study design and lack of a control group. Also, a possible patient selections bias cannot be excluded, as all participants were referrals to our specialized allergy center and often suffer from severe or refractory CIU. 

In conclusion, our data underline the tremendous importance to raise awareness of the psychosocial comorbidities and treatment gaps in CIU in order to improve clinical care in the future. 

## Acknowledgment 

Clinician Scientist Programm, Universitätsmedizin Leipzig. 

## Authors’ contributions 

PK and RT were responsible for the concept of the study; all authors were equally involved in the acquisition, analysis and interpretation of data and to paper writing. All authors gave their final, pre-published approval of the version and agree to be accountable for all aspects of the work. 

## Funding 

Grant received from Hautnetz Leipzig/Westsachsen e.V. (registered association) for research on chronic urticaria and comorbidities. 

## Conflict of interest 

JZ received travel grants and congress support from ALK-Abello, TAKEDA, and CSL Behring, lecture fees from Sanofi Genzyme, and a scientific grant from Novartis. PK has received lecture fees and honoraria from Sanofi Genzyme as well as travel grants/congress support from Sanofi Genzyme, ALK-Abello, Novartis, and Galderma. PK has received a scientific grant from Hautnetz Leipzig/Westsachsen e.V. (registered association) for research on atopic dermatitis and comorbidities. RT has received lecture fees Allmirall, Leopharma, Sanofi Genzyme, AbbVie, Novartis, Takeda, ALK-Abello, Viatris, and Pfizer as well as honoraria for advisory boards from Allmirall, Sanofi, ALK-Abello, Leopharma, Novartis; RT received congress support from Takeda and a nonrestricted scientific grant from Sanofi Genzyme. 

**Table 1.** Scores of validated questionnaires-

**Questionnaire**	**Mean score with SD**	**Unsuspicious score**	**Suspicious score**
UCT	9.1 ± 4.4	≥ 12: 28 (34.1%)	0 – 11: 54 (65.9%)
GAD7	6.9 ± 5.2	0 – 9: 60 (73.2%)	≥ 10 (moderate): 21 (25.6%) ≥ 15 (severe): 9 (11.0%)
HADS-A*	10.2 ± 18.0	0 – 7: 47 (57.3%)	8 – 10 (moderate): 15 (18.3%) ≥ 11 (severe): 17 (20.7%)
HADS-D*	9.2 ± 18.2	0 – 7: 53 (64.6%)	8 – 10 (moderate): 13 (15.9%) ≥ 11 (severe): 13 (15.9%)
CES-D	15.9 ± 10.3	0 – 15: 44 (53.7%)	≥ 23: 19 (23.2%)
CU-Q2oL	52.6 ± 19.8		
UAS7	29.5 ± 33.6		

*n = 79 patients. UCT = Urticaria Control Test; GAD-7 = Generalized Anxiety Disorder-7; HADS = Hospital Anxiety and Depression Scale; CES-D = Epidemiologic Studies Depression Scale; CU-Q2oL = Chronic Urticaria Quality Of Life Questionnaire; UAS7 = Urticaria Activity Score.
